# The Polyol Pathway as a Mechanism for Diabetic Retinopathy: Attractive, Elusive, and Resilient

**DOI:** 10.1155/2007/61038

**Published:** 2007-07-30

**Authors:** Mara Lorenzi

**Affiliations:** Schepens Eye Research Institute and Department of Ophthalmology, Harvard Medical School, 20 Staniford Street, Boston, MA 02114, USA

## Abstract

The polyol pathway is a two-step metabolic pathway in which glucose is reduced to sorbitol, which is then converted to fructose. It is one of the most attractive candidate mechanisms to explain, at least in part, the cellular toxicity of diabetic hyperglycemia because (i) it becomes active when intracellular glucose concentrations are elevated, (ii) the two enzymes are present in human tissues and organs that are sites of diabetic complications, and (iii) the products of the pathway and the altered balance of cofactors generate the types of cellular stress that occur at the sites of diabetic complications. Inhibition (or ablation) of aldose reductase, the first and rate-limiting enzyme in the pathway, reproducibly prevents diabetic retinopathy in diabetic rodent models, but the results of a major clinical trial have been disappointing. Since then, it has become evident that truly informative indicators of polyol pathway activity and/or inhibition are elusive, but are likely to be other than sorbitol levels if meant to predict accurately tissue consequences. The spectrum of abnormalities known to occur in human diabetic retinopathy has enlarged to include glial and neuronal abnormalities, which in experimental animals are mediated by the polyol pathway. The endothelial cells of human retinal vessels have been noted to have aldose reductase. Specific polymorphisms in the promoter region of the aldose reductase gene have been found associated with susceptibility or progression of diabetic retinopathy. This new knowledge has rekindled interest in a possible role of the polyol pathway in diabetic retinopathy and in methodological investigation that may prepare new clinical trials. Only new drugs that inhibit aldose reductase with higher efficacy and safety than older drugs will make possible to learn if the resilience of the polyol pathway means that it has a role in human diabetic retinopathy that should not have gone undiscovered.

## 1. INTRODUCTION

The question of whether the minor pathway of glucose metabolism, called the polyol pathway, is an important player in retinopathy and other complications of human diabetes has been asked for over three decades [1], and the answer is not yet in. Such state of things begets two questions: why do we not have an answer yet? and perhaps more pointedly, why is the question still alive?

The answer to the first question begins as direct and practical, but becomes interlocutory. We have not had probes to address rigorously the question of whether the polyol pathway has a pathogenic role in human diabetic retinopathy. In practical terms, we have not had available drugs with a high therapeutic index in humans, that is, effective and well tolerated at the same time, so as to make possible their usage at doses documented to inhibit the pathway fully and predictably in the tissues of interest. But do we know which is the gold standard by which to measure “inhibition of the polyol pathway”? We are becoming aware that such knowledge is pivotal, and not easy to acquire. The answer to why we are still courting and querying the polyol pathway has to do with both the current treatment of diabetes and the polyol pathway itself. Intensive glycemic control is clearly effective in reducing the incidence and progression of diabetic retinopathy [2, 3], but with the means available today even the best efforts do not achieve normal glucose homeostasis, and retinopathy and other complications continue to develop and progress to clinically important stages also among well-controlled patients [2–4]. We are not yet able to offer adjunct treatments that can preempt the damaging effects of the residual hyperglycemia. The polyol pathway is by all criteria an enormously attractive target for adjunct treatment. The polyol pathway is also enormously resilient, and just when investigators try to put it aside, it scores new points and returns to the fore.

Over three decades, much has been written on the polyol pathway and the complications of diabetes, and much has been captured in informative reviews [5, 6]. My goal in this writing is to extract from the existing body of knowledge what justifies a continuous interest in the pathway from the standpoint of human diabetic retinopathy, and to highlight actions needed to give the pathway a role or a dismissal.

## 2. THE POLYOL PATHWAY IS A PLAUSIBLE BIOCHEMICAL MECHANISM FOR DIABETIC RETINOPATHY

The polyol pathway of glucose metabolism becomes active when intracellular glucose levels are elevated [1, 5]. Aldose reductase (AR), the first and rate-limiting enzyme in the pathway, reduces glucose to sorbitol using NADPH as a cofactor; sorbitol is then metabolized to fructose by sorbitol dehydrogenase that uses NAD^+^ as a cofactor. The effects are several. Sorbitol is an alcohol, polyhydroxylated, and strongly hydrophilic, and therefore does not diffuse readily through cell membranes and accumulates intracellularly with possible osmotic consequences [1]. (Production of intracellular osmolytes to counterbalance extracellular hypertonicity is a likely physiological role of AR in the kidney medulla [7].) The fructose produced by the polyol pathway can become phosphorylated to fructose-3-phosphate [8, 9], which is broken down to 3-deoxyglucosone; both compounds are powerful glycosylating agents that enter in the formation of advanced glycation end products (AGEs) [8]. The usage of NADPH by AR may result in less cofactor available for glutathione reductase, which is critical for the maintenance of the intracellular pool of reduced glutathione (GSH). This would lessen the capability of cells to respond to oxidative stress [10]. Compensatory increased activity of the glucose monophosphate shunt, the principal supplier of cellular NADPH, may occur [10]. The usage of NAD by sorbitol dehydrogenase leads to an increased ratio of NADH/NAD^+^, which has been termed “pseudohypoxia” and linked to a multitude of metabolic and signaling changes known to alter cell function [11]. It has been proposed that the excess NADH may become a substrate for NADH oxidase, and this would be a mechanism for generation of intracellular oxidant species [12]. Thus, activation of the polyol pathway, by altering intracellular tonicity, generating AGEs precursors, and exposing cells to oxidative stress perhaps through decreased antioxidant defenses and generation of oxidant species, can initiate and multiply several mechanisms of cellular damage.

Retinal ganglion cells, Müller glia, and vascular pericytes and endothelial cells are endowed with aldose reductase in all species studied, including humans (reviewed in [13]). Hence, these cell types are exposed to polyol pathway activation in diabetes. These are also the cells that manifest the best-known changes or damage in diabetes [14]. The biochemical consequences of polyol pathway activation have been studied in the whole retina of diabetic animals. The best-documented are the accumulation of sorbitol and fructose [15, 16], and the generation or enhancement of oxidative stress. The retina of experimentally diabetic rats shows increased lipid peroxidation products [16], increased nitrotyrosine [17], and depletion of antioxidant enzymes [16]. These abnormalities are prevented by drugs that inhibit AR [15–17]. Insofar as indices of polyol pathway-induced oxidative stress are measurable in preparations of the whole retina, the abnormalities are likely to occur in most cell types or at least in cells that are highly represented in the whole retina. Müller glial cells are candidates because they are large cells present in high number in the retina [18]. We cannot exclude that other mechanisms of polyol pathway-induced damage may be operative in selected types of retinal cells. For example, osmotic stress seemingly cannot be invoked from data in the whole retina because retinal levels of sorbitol increase in diabetic rats only 3–8 fold above control [15, 16], and are far from the concentrations that could induce osmotic stress [1]. However, a cell type that had an especially high ratio of AR to sorbitol dehydrogenase could accumulate sorbitol with intracellular consequences, and yet the amount would be diluted substantially in measurements taken in the whole retina. Investigation that compares and contrasts the kinetics and consequences of polyol pathway activation in the relevant retinal cell types—neurons, Müller glia, pericytes, and endothelial cells—might therefore generate important new insights. At this time, the information from the biochemical measurements and the dose-response studies with ARIs point to oxidative stress as the strongest candidate mechanism for polyol pathway-dependent cellular damage in the diabetic retina.

## 3. THE POLYOL PATHWAY IS A CONTRIBUTOR TO EXPERIMENTAL DIABETIC RETINOPATHY

Two waves of studies addressed the role of the polyol pathway in experimental diabetic retinopathy. A first wave of studies centered on the galactosemic model. In the early 80's, Engerman and Kern described the diabetic-like retinopathy of dogs fed a diet rich in galactose [19], and Robison et al. reported that the basement membrane thickening seen in the retinal capillaries of rats fed a diet rich in galactose was prevented by an AR inhibitor (ARI) [20]. The observation that galactosemia, that is, the isolated elevation of a hexose in blood without other hormonal or metabolic abnormalities [19], could mimic most features of the retinal microangiopathy caused by diabetes is, to date, the most specific indictment of the role of hyperglycemia in causing retinopathy in diabetes. (The design of the Diabetes Control and Complications Trial [DCCT], where intensive insulin treatment ameliorated the diabetic state comprehensively, cannot isolate precisely the benefits of correcting hyperglycemia, and thus the role of hyperglycemia in the complications.) The galactosemic model was highly relevant to the polyol pathway because the set of enzymes necessary to permit the entry of dietary galactose into the glycolytic pathway is present only in the liver [21]. In congenital disorders where enzymes of galactose metabolism are defective, blood galactose levels increase and many peripheral tissues accumulate two metabolites, galactose-1-phosphate, and galactitol [21]. Galactitol is produced from galactose via the action of AR; and the AR from all species studied—human, dog, and rat—displays a lower Km for galactose than for glucose [22]. An ARI, given at a dose of 65 mg/Kg/day prevented the diabetic-like retinal vascular histopathology developed by rats fed a 50% galactose diet [23]. However, the full complement of retinal microangiopathy developed by dogs fed a 30% galactose diet was only delayed [24] or not prevented at all [25] by the ARI sorbinil. The dose of sorbinil used in the galactose-fed dogs (60 mg/Kg/day on average) and devised to inhibit accumulation of galactitol in erythrocytes was not different from the dose used in the galactose-fed rats. One group of investigators noted, however, that sorbinil was metabolized more rapidly in dogs than in rats, yielding unexpectedly short plasma half-life [24]. The same group also examined retinal changes in the galactose-fed dog model treated with two doses of a sorbinil analog, and found evidence for dose-dependency of the beneficial effects of ARI treatment [26].

In one of the above studies, experimentally diabetic dogs were studied alongside galactose-fed dogs [25]. To diabetic dogs, sorbinil was administered in a lesser dose (20 mg/Kg/day) than to galactosemic dogs, targeting inhibition of diabetes-induced sorbitol accumulation in erythrocytes. The dose yielded 90% sorbitol inhibition in erythrocytes as well as retina. However, sorbinil did not prevent the development of retinal microangiopathy in the diabetic dogs [25]. Shortly before, the final results of the sorbinil retinopathy trial had become available. The trial had tested sorbinil at a dose of 250 mg/day (the equivalent of 3.5 mg/Kg/day in a 70 Kg individual), administered for 3 years to patients with type 1 diabetes with absent or very mild retinopathy. The data indicated that sorbinil did not have a clinically important effect on the course of human diabetic retinopathy [27]. Interpretative caveats apply to these negative results, as discussed later in this writing. Nonetheless, at the time the results understandably dampened the enthusiasm in pursuing the polyol pathway as a major player and target in diabetic retinopathy.

However, when new features of diabetic retinopathy became known in the late 90's, the polyol pathway commanded a second wave of investigations, showing its resilience. We developed an interest in the polyol pathway because it was the rational first choice for a new mechanistic question. We wished to understand the mechanism for the reactive characteristics of Müller glial cells [28, 29] and apoptosis of neurons (mostly ganglion cells) [30] occurring early in both human and experimental diabetic retinopathy; and Müller and ganglion cells are the retinal cell types most consistently found to contain aldose reductase in humans, rats, and dogs [31–34]. In view of the fact that sorbinil had failed to prevent the lesions of retinopathy in diabetic dogs when used at a dose of 20 mg/Kg/day [25], we selected the larger dose used in the galactosemic models. We observed that, in the retina of rats with 2.5 months of diabetes, neurons undergoing apoptosis indeed contained aldose reductase, and that sorbinil 65 mg/Kg/day prevented neuronal apoptosis, the prominent increase in glial fibrillary acidic protein (GFAP) in Müller cells indicating a reactive state, and changes in astrocytes [15].

The question arose whether in the diabetic rat also the vascular abnormalities of retinopathy receive a contribution from the polyol pathway. ARIs had been noted to prevent hemodynamic changes and vascular permeability changes in rats with 6 weeks of diabetes [35] and retinal capillary basement membrane thickening in rats with 6 months of diabetes [36]. Surprisingly, there were however no published studies addressing, in diabetic rats, the effect of aldose reductase inhibition on the ultimate manifestations of retinal microangiopathy in diabetes, apoptosis of pericytes and endothelial cells, and development of acellular capillaries. We had shown that microvascular cell apoptosis precedes [37] and predicts [38] the development of acellular capillaries, which is in turn a key event in diabetic retinopathy [39, 40] because it heralds retinal ischemia and, in humans, transition to sight-threatening proliferative retinopathy. We thus view the development of acellular capillaries as the required read-out for any modeling, pathogenetic construct, or intervention related to the microangiopathy of diabetic retinopathy. We went on to perform the studies addressing the role of the polyol pathway, and we targeted vascular abnormalities that we had documented to occur also in the human diabetic retina [37, 41]. The retinal vessels of diabetic rats treated with sorbinil for the 9-month duration of diabetes showed prevention of (i) early complement activation, (ii) decreased levels of complement inhibitors, (iii) microvascular cell apoptosis, and (iv) acellular capillaries [13]. Also other investigators had reported sparing of pericytes by treatment with an ARI other than sorbinil in rats with 15 months of diabetes [42]. (In this particular study the pericyte counts were performed in retinal sections, specimens that are suboptimal for the purpose.) Insofar as the pericytes and endothelial cells of rat retinal vessels contain AR [13], it is plausible that the ARIs prevented the microangiopathy by inhibiting polyol pathway directly in vascular cells.

The diabetic mouse has contributed information that supports the role of the polyol pathway in diabetic retinopathy, but has generated an important question as yet unanswered. We compared and contrasted the mouse to the rat because of the known differences in polyol pathway activation between these species. The mouse is known to have, in the lens, one-tenth of the aldose reductase activity present in the rat lens [43], and its extreme resistance to cataract under conditions of hyperglycemia [43] and galactosemia [44] can be overcome only by introduction of a human aldose reductase transgene [44, 45]. We reasoned that the mouse may have low aldose reductase activity also in the retina. In fact, C57BL/6J mice, rendered diabetic with streptozotocin and exhibiting hyperglycemia as severe as diabetic rats, did not accumulate sorbitol or fructose in the retina [15]. Obrosova et al. made similar observations, and reported additional and important biochemical differences between the retinas of diabetic mice and diabetic rats [46]. The diabetic mice did not show apoptosis of ganglion cells or reactivity of Müller glial cells, and this was consistent with the evidence in diabetic rats that polyol pathway activation is the inducer of the neuroglial abnormalities. A different group of investigators confirmed the absence of neuronal apoptosis and Müller cell reactivity in C57BL/6J mice studied up to one year-duration of streptozotocin-diabetes [47]. Two groups reported instead loss of retinal neurons in diabetic mice, one group in the streptozotocin model [48] and the other in the Ins2^AKITA^ mouse maintained on the C57BL/6J background [49]. These reports did not present counts of apoptotic cells; the conclusions on neuronal death were based on assays for activated caspase 3 and/or morphometry of retinal layers. Nor did the studies investigate retinal polyol pathway activity. However, the Ins2^AKITA^ mouse was specifically reported not to show Müller cell reactivity [49], in agreement with the findings in the streptozotocin diabetic mouse [15, 47]. Recent observations in *db*/*db* mice, which are a model for type 2 diabetes, indicate that very long duration of diabetes (15 months) leads to increased expression of retinal AR, Müller cell reactivity, neural cell apoptosis, and vascular changes; and that these abnormalities do not appear in *db*/*db* mice rendered genetically AR-deficient [50]. Thus, in diabetic mice, retinal neuroglial abnormalities are not found as consistently as in diabetic rats. When neuroglial abnormalities are undetectable, polyol pathway activity is likewise undetectable. When neuroglial abnormalities are present, polyol pathway activity appears to be the inciting cause.

The question generated by the diabetic mouse and as yet unanswered is the relationship of acellular capillaries to the polyol pathway in this model. The streptozotocin-diabetic C57BL/6J mouse model develops a progressively greater number of acellular capillaries with increasing duration of diabetes, and the acceleration over control begins after 6 months of diabetes [47], when neither retinal accumulation of polyols nor neuroglial abnormalities can be demonstrated [15, 47]. Does this indicate that in the diabetic mouse retinal vascular cells activate the polyol pathway and are damaged by the consequences, when neural and glial cells do not, or not as yet? Because vascular cells represent only a minor contribution to total retinal cellularity, their accumulation of polyols would go unnoticed in preparations of whole retina. Unfortunately, the studies in the AR-null *db*/*db* mice could not answer the question, because the development of acellular capillaries was not addressed specifically. (In that model, vessels were actually reported to be present in larger number than in nondiabetic mice [50]. An important interpretative caveat is that the vessels were counted as dots or tubes staining for IgG on retinal sections; and the number of such images can be influenced by several confounders, such as the plane and thickness of sections, as well as vessel tortuosity and the state of vessel perfusion.)

To summarize the observations in animal models, there is weighty evidence for the concept that polyol pathway activation is a sufficient mechanism for the retinal abnormalities induced by diabetes in the rat. One missing piece of evidence precludes a similar comprehensive conclusion in the diabetic mouse. The results from the diabetic dog studies and the sorbinil trial do not support the importance of the polyol pathway in the development of diabetic retinopathy in the dog model or in humans. But these latter experiments are the older, and it is becoming apparent that, if designed today, they would be designed differently. We examine the issues in the following sections.

## 4. DO WE KNOW HOW TO SILENCE THE POLYOL PATHWAY WHEN THE GOAL IS PREVENTION OF TISSUE DAMAGE?

The polyol pathway is, at first glance, a dream target when aiming to develop drugs for prevention of the complications of diabetes. The rate-limiting enzyme of the pathway, AR, acts on the glucose molecule, and therefore at the most upstream possible site in the cascade of glucose toxicity. AR is encoded by one gene [5, 51], has a known structure and kinetic properties [52], and its activity is inhibitable by multiple classes of small molecule compounds [53]. Complete AR deficiency produces only a mild nephrogenic diabetes insipidus [54]. On this basis, AR inhibition appears rational, feasible, and benign; and if we could just add to the tools a reliable indicator of AR inhibition permitting us to target and monitor in tissues the therapeutic effects of ARIs, we could readily implement rigorous preclinical studies and clinical trials.

However, the “reliable indicator of AR inhibition” is turning out to be an elusive target, and the reason is that we do not know for certain how polyol pathway activity causes tissue damage. What we know is that when ARIs have been dosed to prevent sorbitol accumulation, as in the studies with diabetic dogs or the sorbinil trial (where sorbitol was monitored in red blood cells, and not even normalized there), very little tissue benefit has ensued. When ARIs have been used in larger doses targeted to prevent the accumulation of fructose rather than sorbitol, they have been more successful. The concordant dose-dependency for effects on fructose and tissue abnormalities has been demonstrated directly in the nerve [55], and was also seen clearly in the retina. When we tested ARIs on diabetic retinopathy in the rat, we used a 65 mg/kg/day sorbinil dose [13, 15] that was shown in preliminary experiments to inhibit both sorbitol and fructose formation. The greater relevance of fructose than sorbitol as measure of polyol pathway activity is easily understandable when thinking that sorbitol accumulation may be minuscule under conditions in which AR is very active if also sorbitol dehydrogenase is very active. Under such conditions, sorbitol levels may never increase and yet the pathway will have processed a large amount of glucose, and the flux of glucose will have used NADPH and generated NADH and fructose. These may ultimately be the more relevant abnormalities for cells and tissues. Oates et al. are currently showing that in diabetic rat nerve the ratio of oxidized glutathione (GSSG) to reduced glutathione (GSH) increases, indicating oxidative stress; and that normalization of the ratio requires an even larger dose of ARI than the normalization of fructose, at least in acute experiments [56]. The implication of these studies is very important: if we must limit the oxidative consequences of the polyol pathway in order to limit its cellular toxicity, we may need to inhibit AR much more drastically, at least to the degree achieved in recent rat studies [13, 15–17], and such inhibition required doses of ARIs twenty times larger than those used in the sorbinil trial.

A noteworthy issue with ARIs is that they can inhibit also aldehyde reductase, another enzyme in the aldo-keto reductase superfamily that plays a role in the detoxification of reactive aldehydes [22, 57, 58]. All ARIs inhibit AR more than aldehyde reductase, but some ARIs such as sorbinil do not have a high degree of selectivity for AR versus aldehyde reductase [22, 53, 57]. This generates the question of whether some inhibition of aldehyde reductase contributes to the beneficial effects of ARIs, especially at the higher doses; or, conversely, contributes unwanted side effects. We recently had the opportunity to test an ARI from an entirely new structural class, a sulfonylpyridazone characterized as one of the most potent and selective ARIs yet described. In particular, the IC50 of ARI-809 (compound 19 m in [53]) for aldehyde reductase is 930 nM as compared to 1 nM for AR [53]. Such selectivity permitted us to target critically the role of the polyol pathway in the early stages of the development of experimental diabetic retinopathy. ARI-809 administered to diabetic rats at doses documented to inhibit both sorbitol and fructose accumulation in the retina, improved survival, inhibited cataract development, and protected the retina from all early neuronal, glial, and vascular abnormalities known to also occur in human diabetes [59]. On this basis, it can be stated that aldose reductase is itself the key relay that converts hyperglycemia into glucose toxicity for specific cell types in the retina. Whether ARI-809 or other highly specific ARIs will also help address the problem of toleration and side effects will need to be addressed in humans.

If further experiments confirm that only large ARI doses can block the polyol pathway to the extent required to prevent tissue damage, we are clearly poorly equipped to bring the concept to test in humans. The two chemical classes of ARIs that have been mostly tested in phase III trials, the carboxylic acid inhibitors (e.g., zopolrestat) and the spirohydantoin inhibitors (e.g., sorbinil), have a suboptimal therapeutic index. Both classes have shown side effects: liver and/or renal toxicity in the former, and potentially serious hypersensitivity reactions in the latter [27]. The different type of side effects suggests that they are not consequences of AR inhibition, but rather of other properties of the individual drugs. Nonetheless, the side effects would preclude any escalation of the doses tested to date. There are new ARIs of the spiroimide/hydantoin class that show greater potency than sorbinil, and have achieved in diabetic patients a robust inhibition of the polyol pathway in sural nerves [60], as well as improvement in signs and symptoms of sensorimotor polyneuropathy at well-tolerated doses [61–63]. The results of larger trials with these drugs are expected soon.

## 5. IS THE POLYOL PATHWAY IMPORTANT IN HUMAN DIABETIC RETINOPATHY? AND WHY SHOULD WE CARE TO KNOW?

The results of the sorbinil retinopathy trial could have been a false negative or a true negative. A false negative could have occurred for at least two reasons. The first is that the dose of sorbinil may have been insufficient to achieve and sustain inhibition of retinal polyol pathway; we found that much larger doses—not usable in humans—were needed to achieve prevention of retinal polyol pathway activation in the diabetic rat [13, 15]. When the intent is to test a pathogenic mechanism for disease, there is a stringent requirement for documentation that the chosen intervention inhibits or attenuates the target mechanism at the sites of interest. The DCCT and United Kingdom Prospective Diabetes Study which set out to test the role of metabolic control in the complications of diabetes implemented laborious strategies to verify before the trial, and document extensively throughout the trial, that two levels of glycemic control could be achieved and maintained, and that they were sufficiently apart in a sufficient number of study participants to permit testing the hypothesis. Uncertainty about whether a drug or intervention is delivered in sufficient dose to achieve effects at target tissue is a recurrent issue in clinical trials. It continues to be one of the most commonly proposed mechanisms to explain discrepancies between encouraging preclinical studies and inconclusive trials. Just recently, it was discussed with regard to the benefits of insulin-like growth factor-1 in motor neuron diseases [64] and exogenous surfactant replacement in the acute respiratory distress syndrome [65]. The second reason for which the sorbinil trial could have yielded false negative results is that it was an intervention trial, where approximately half of the study population had clinical evidence of retinopathy, albeit mild [27]. Interventions do not work as well as prevention in diabetic retinopathy [2]. This is likely to be especially true for interventions such as ARIs that target early events in the cascade that leads to the demise of retinal vascular cells.

It remains possible that the results of the sorbinil trial were a true negative, and it appears wise to investigate how solid is the rationale for advocating new rigorous trials. Information gathered by studying post-mortem eyes is consistent with activity of the polyol pathway in human diabetes. Retinas from diabetic patients with retinopathy show more abundant AR immunoreactivity in ganglion cells, nerve fibers, and Müller cells than retinas from nondiabetic individuals [66]. In our experiments, human retinas from nondiabetic eye donors exposed to high glucose levels in organ culture accumulate sorbitol to the same extent as similarly incubated retinas of nondiabetic rats, as well as retinas dissected from diabetic rats [13]. The comparable biochemical outcome of AR activity in the human and rat retinas underscores that human AR is readily responsive to hyperglycemia. The human enzyme is in fact widely used as a transgene to make the mouse susceptible to diabetic complications, from cataract [45] to atherosclerosis [67]. It is thus apparent that cells in the human retina—including vascular endothelial cells and pericytes [13]—can have an active polyol pathway in the presence of hyperglycemia.

An additional type of new information that justifies revisiting the role of the polyol pathway in human diabetic retinopathy is the finding that several polymorphisms in the promoter region of the AR gene are associated with susceptibility to, or more rapid progression of, diabetic retinopathy (reviewed in [51]). Of particular interest has been the Z−2 allele of the (AC)n dinucleotide repeat sequence located 2.1 kb upstream of the AR transcription start site [68]. The Z−2 allele is in linkage disequilibrium with another polymorphism in the AR promoter that also increases susceptibility to retinopathy [69]; and appears to be associated with higher levels of AR mRNA [70]. Of much interest, the association of the Z−2 allele with increased AR expression was found only in diabetic patients, not among nondiabetic individuals [70], suggesting an interaction with diabetes/hyperglycemia. Although the best-known type of interaction of hyperglycemia with AR is to provide substrate for activity of the enzyme (which has a high Km for glucose), there are reports indicating also an effect of hyperglycemia on AR gene expression [71, 72]. It can be expected that such effect would increase the magnitude of polyol pathway activation and its tissue consequences.

However, the ultimate question is not whether further investigation of the polyol pathway in diabetic retinopathy is justified, but rather whether it is needed. After all, the polyol pathway is not the only candidate mechanism for diabetic retinopathy on which to base the development of adjunct treatments. In experimentally diabetic animals several interventions targeting pathogenic mechanisms other than the polyol pathway have proven to be capable of inhibiting the development of retinal acellular capillaries—aminoguanidine [38, 73] and pyridoxamine [74] given to inhibit AGE formation, benfotiamine [75] given to divert glycolytic intermediates from pathways of hyperglycemic damage, the poly(ADP-ribose) polymerase (PARP) inhibitor PJ-34 [76] given to counteract the potentially proinflammatory action of PARP, and aspirin [77, 78] given at doses that have antiplatelet and anti-inflammatory effects. The first three compounds target upstream consequences of hyperglycemia. Even if some AGEs precursors are generated through polyol pathway activation [8, 9, 11], it is actually encouraging that drugs that inhibit AGE formation without having any effect on AR such as aminoguanidine [38] can prevent the development of acellular capillaries. PARP inhibitors and aspirin are likely to work on downstream events. The fact that removing one upstream or downstream contribution to the development of acellular capillaries lessens or delays considerably their development indicates that there is interaction and reinforcement among the causative events triggered by diabetes. If such concept applies also to human retinopathy, it would justify the development of multiple adjunct drugs of different classes, thus able to accommodate issues of sensitivity and tolerance by individual patients, and usable in combinations that may maximize efficacy and safety.

Recent findings, however, indicate that some consequences of the polyol pathway may be preventable only by ARIs. We contrasted the effects of aspirin, clopidogrel (a selective antiplatelet agent), and sorbinil on the comprehensive picture of diabetic retinopathy, and found that both aspirin and sorbinil succeeded in preventing the acellular capillaries, but only sorbinil prevented the reactive phenotype of Müller cells [78]. We obtained identical results using the structurally novel and highly selective ARI-809 [59]. To our knowledge, ARIs are the only type of drugs shown to date to prevent the Müller cell reactivity that occurs in the diabetic rat retina, and that is also characteristic of human diabetic retinopathy [28]. We do not know yet whether and where the Müller cell reactivity is important in the process of diabetic retinopathy, but the arguments we see in support of this possibility have prompted us to begin experimental testing. Müller cells have essential roles in the whole retina, from structural support to neurotransmitter metabolism [18], and uniquely specialized roles in the inner retina—formation and maintenance of the blood-retinal barrier, and dehydration of the interstitium by pumping water into the capillaries [79, 80]. In primates, the Müller cell density is over 5 times greater in the parafoveal than in peripheral regions of the retina [18]. These characteristics make dysfunctional Müller cells relevant to the development of macular edema, a complication of diabetic retinopathy that cannot be studied in the usual animal models, because rodents do not have a macula. In human diabetes, macular edema is characterized by the accumulation of extracellular fluid in Henle's layer and the inner nuclear layer of the retina, and the clinically important accumulation is thought to require not only increased influx of water from the abnormally permeable capillaries, but also a defect in the reabsorptive mechanism. The Müller cells *are* such reabsorbing mechanism for the inner retina [80]. Diabetic macular edema is strongly associated with poor glycemic control [reviewed in [81]]. Poor glycemic control means for Müller cells high intracellular glucose because they have mostly the insulin-independent GLUT-1 glucose transporters [82], and high levels of polyol pathway activity on account of the especially abundant AR levels. This combination may expose the cells to all consequences of the pathway including oxidative and osmotic stress.

On the basis of the biochemical data obtained in the whole retina [13, 15, 16, 46], we expect that Müller cells experience oxidative stress from activation of the polyol pathway, and that this triggers the reactive phenotype. However, if Müller cells were to experience in human diabetes also osmotic consequences of the polyol pathway, there would be two novel implications for human diabetic retinopathy. The first would be the identification of a discrete biochemical mechanism for the cystoid features of macular edema in diabetes, because swelling of Müller cells themselves is at the basis of cystoid edema [80]. The second implication would be that no other drugs but ARIs could be expected to correct this particular mechanism for Müller cell abnormalities. We know that diabetes induces a large number of changes in gene expression in Müller cells indicating both a reactive phenotype and altered functions [83], and the nature of the changes suggests multiple inducing mechanisms. It is thus likely that prevention of the spectrum of Müller cell abnormality does require the high doses of ARIs we tested [15, 59, 78], but it is possible that osmotic consequences of polyol pathway activation may be approachable with lesser doses. Then, ARIs would have three different indications related to diabetic retinopathy—a first and unique one directed at the acute relief of macular edema, a second one directed at the prevention of both macular edema and microangiopathy, and a third one directed at the prevention of macular edema in the context of a drug combination where the other drug targets the microangiopathy. In view of the different duration of treatment required by the different indications and the different specific targets, the three indications could be served by ARIs that differ in potency as well as therapeutic index. New experiments will now address these speculative possibilities.

## 6. CONCLUSIONS

The latest wave of studies on the polyol pathway has given us a more complete picture of what the pathway *can* do in the human retina, and how. The polyol pathway can be active in the human retina; it can be active in endothelial cells and not just in pericytes; it can be the mechanism for the changes we see in the Müller cells of human diabetic retinas; through activity in endothelial and Müller cells the pathway can be a strong contributor to the disturbed fluid homeostasis that occurs in the diabetic retina and leads at times to macular edema; through activity in endothelial cells and pericytes, the pathway can be a strong contributor to the vascular cell apoptosis that eventually results in acellular capillaries and retinal ischemia. The preferred mechanism of cellular damage by the polyol pathway appears to be the induction of oxidative stress, although other mechanisms should not be ignored. Interventions with ARIs are successful when the doses used reduce the flux through the pathway and indices of oxidative stress.

In order to move forward and ascertain if what can happen *does in fact happen*, several needs must be met. The first is to have available new ARIs, best if more than one chemical type, combining higher levels of efficacy, selectivity, and safety in humans than those exhibited by older drugs. The pharmaceutical industry may need to apply to the problem some new thinking, especially in view of the fact that ARIs could become the “one drug-option” for diabetic retinopathy only if usable at doses that inhibit AR and flux through the pathway more substantially than the older drugs. The second need is clarification of whether distinct cell types in the retina are exposed to specific consequences of, or have specific susceptibilities to, the different mechanisms of cellular damage initiated by the polyol pathway. Satisfying these two needs may yield means with which to satisfy the third, which is the identification of reliable indicators of polyol pathway activity. Indicators are necessary in order to learn whether the polyol pathway is in fact active in the human diabetic retina, and how best to inhibit the pathway with the new ARIs in preparation for longer trials. In general, these needs are not different from those faced by all other candidate mechanisms and drugs seeking a role in human diabetic retinopathy [84]. We all look forward to bringing one or more adjunct treatment to clinical reality, and may ultimately welcome the resilience of the polyol pathway if we were to find that it has unique roles and/or it offers unique solutions to human diabetic retinopathy.
